# Selinexor plus low-dose dexamethasone in Chinese patients with relapsed/refractory multiple myeloma previously treated with an immunomodulatory agent and a proteasome inhibitor (MARCH): a phase II, single-arm study

**DOI:** 10.1186/s12916-022-02305-4

**Published:** 2022-04-05

**Authors:** Lugui Qiu, Zhongjun Xia, Chengcheng Fu, Wenming Chen, Chunkang Chang, Baijun Fang, Gang An, Yongqiang Wei, Zhen Cai, Sujun Gao, Jianyu Weng, Lijuan Chen, Hongmei Jing, Fei Li, Zhuogang Liu, Xiequn Chen, Jing Liu, Aihua Wang, Yang Yu, Wenxi Xiang, Kevin Lynch, Zhinuan Yu, Weijun Fu

**Affiliations:** 1 National Clinical Research Center for Blood Diseases, State Key Laboratory of Experimental Hematology, Institute of Hematology and Blood Diseases Hospital, Chinese Academy of Medical Sciences and Peking Union Medical College, Tianjin, 300020 China; 2 Department of Hematology, Sun Yat-sen University Cancer Center, Guangzhou, China; 3 The First Affiliated Hospital of Soochow University, Suzhou, China; 4 Department of Hematology, Beijing Chao-Yang Hospital, Capital Medical University, Beijing, China; 5 Department of Hematology, Shanghai Jiao Tong University Affiliated Sixth People’s Hospital, Shanghai, China; 6 Department of Hematology, Henan Institute of Hematology, Henan Cancer Hospital, The Affiliated Cancer Hospital of Zhengzhou University, Zhengzhou, China; 7 Department of Hematology, Nanfang Hospital, Southern Medical University, Guangzhou, China; 8 Bone Marrow Transplantation Center, Department of Hematology, The First Affiliated Hospital, Zhejiang University School of Medicine, Hangzhou, China; 9 Department of Hematology, the First Affiliated Hospital of Jilin University, Changchun, China; 10 Department of Hematology, Guangdong Provincial People’s Hospital, Guangzhou, China; 11 Department of Hematology, Jiangsu Province Hospital, The First Affiliated Hospital with Nanjing Medical University, Nanjing, China; 12 Department of Hematology and Lymphoma Research Center, Peking University Third Hospital, Beijing, China; 13 Department of Hematology, the First Affiliated Hospital of Nanchang University, Nanchang, China; 14 Department of Hematology, Shengjing Hospital of China Medical University, Shenyang, China; 15 Department of Hematology, Xi Jing Hospital affiliated to the Fourth Military Medical University, Xi’an, China; 16 Department of Hematology, the Third Xiangya Hospital of Central South University, Changsha, China; 17 Antengene Corporation Co., Ltd, Shanghai, China; 18 Department of Hematology, Changzheng Hospital, Shanghai, 200003 China; 19 Department of Hematology, Shanghai Fourth People’s Hospital, School of Medicine, Tongji University, Shanghai, 200434 China

**Keywords:** Multiple myeloma, Relapsed and refractory, Selinexor, Chinese patients

## Abstract

**Background:**

Selinexor 80 mg combined with low-dose dexamethasone (Sd) demonstrated significant clinical benefit in patients with relapsed/refractory multiple myeloma (RRMM) who had disease refractory to a proteasome inhibitor (PI), an immunomodulator (IMiD), and an anti-CD38 monoclonal antibody based on a global phase II STORM study. The present study, MARCH, addresses China regulatory needs to further validate the data from STORM in Chinese patients with RRMM.

**Methods:**

The MARCH study was conducted at 17 sites in China, where eligible Chinese RRMM patients who had disease refractory to PI and IMiD were enrolled. Selinexor 80 mg combined with dexamethasone 20 mg was administered orally on day 1 and day 3 of each week in 4-week cycles. The primary endpoint was the overall response rate (ORR) per an independent review committee, with the null hypothesis of ≤15%. Patients who received at least 1 dose of study treatment were included in the safety population. The pharmacokinetic (PK) profile was characterized by parameter and ethnicity sensitivity analyses.

**Results:**

A total of 82 patients with RRMM were enrolled in the study, with a median age of 60 years. Of the 82 patients, 55 patients (67.1%) had high-risk cytogenetic abnormalities, defined as one or more of del 17p13, t(4;14), t(14;16), or 1q amplification identified by fluorescence in situ hybridization (FISH); 18 patients (22.0%) had abnormal renal function. Enrolled patients were heavily pre-treated with a median prior regimen number of 5. All 82 patients (100%) were refractory to both PI and IMiD, including 20 patients (24.4%) categorized as triple-class refractory population (refractory to PI, IMiD, and daratumumab). Ten patients (12.2%) had undergone CAR-T therapy. ORR was 29.3% (95% *CI* 19.7, 40.4) with a median DOR of 4.7 months. The median PFS and OS were 3.7 and 13.2 months, respectively. ORR was 25.0% (95% *CI* 8.7, 49.1) in the triple-class refractory population. Efficacy was consistent across various subgroups. The most frequent grade 3/4 adverse events (AEs) included anemia (57.3%), thrombocytopenia (51.2%), lymphopenia (42.7%), neutropenia (40.2%), hyponatremia (29.3%), and lung infection (26.8%). Serious AEs were reported in 54.9% of patients. No significant drug accumulation was shown following multiple administrations. No human PK ethnicity difference was identified between Chinese and western patients.

**Conclusions:**

With an encouraging ORR, the MARCH study has demonstrated that selinexor combined with low-dose dexamethasone (Sd) delivers meaningful clinical benefit to Chinese patients with RRMM, including triple-class refractory patients. AEs were expected and manageable with supportive care and dose modification.

**Trial registration:**

ClinicalTrials.gov, NCT03944057 (May 09, 2019); Chinadrugtrials.org.cn, CTR20190858 (June 05, 2019)

**Supplementary Information:**

The online version contains supplementary material available at 10.1186/s12916-022-02305-4.

## Background

Multiple myeloma (MM) is the second most common hematological malignancy worldwide [1]. Though the incidence is relatively low in Asia, it has increased rapidly in this region, with the fastest increase seen in East Asia (including China), where the rate has risen by 262% over the past 26 years [2]. It was estimated that there were 16,500 new MM patients and 10,300 deaths in mainland China in 2016. The age-standardized incidence rate and mortality rate per 100,000 population were 1.03 and 0.67 in 2016 [3].

The introduction of therapies such as proteasome inhibitors (PIs), immunomodulator (IMiDs), and CD38 monoclonal antibodies has significantly improved the prognosis of MM; however, MM remains incurable, and almost all MM patients eventually experience relapse. Relapsed/refractory MM (RRMM) remains a disease with unmet medical needs that will benefit from new treatment options, especially drugs with a novel mechanism of action.

Selinexor (ATG-010, KCP-330, XPOVIO®), as a novel oral drug, is a slowly reversible and highly selective inhibitor of nuclear export (SINE) against exportin 1 (XPO1). The antitumor activity of SINE compounds is mediated by at least 3 different pathways, involving tumor suppressor protein, oncoprotein, and glucocorticoid receptor (GCR). Preclinical studies have shown that selinexor induces apoptosis in a number of myeloma cell lines and has antitumor activity in animal models [4–8]. With dexamethasone, the synergistic effect is shown in vitro and in vivo through increased nuclear localization of GCR and amplified GCR transcriptional activity [9–11].

The combination of selinexor and low-dose dexamethasone (Sd) was approved by the US Food and Drug Administration based on safety and efficacy data from the STORM study [12]. The Sd regimen demonstrated an ORR of 26.2% in penta-exposed, triple-class refractory RRMM, with manageable toxicities. In view of the outcomes in the STORM study, we initiated MARCH, a bridging study in China with a similar study design, to verify the efficacy, safety, and PK profile of selinexor combined with low-dose dexamethasone in the treatment of Chinese RRMM patients whose disease were refractory to both PI and IMiD.

## Methods

### Study design and participants

The open-label, single-arm, phase II MARCH study was conducted at 17 sites in China. The study addresses Chinese regulatory needs by providing a bridge to the global phase II STORM study, using consistent primary endpoint, eligible criteria, treatment regimens, and disease assessment schedule. STORM eligible patients had previously been treated with lenalidomide, pomalidomide, bortezomib, carfilzomib, and daratumumab. They had disease refractory to at least one IMiD, one PI, daratumumab, and their most recent regimen. In contrast, eligible patients in MARCH had been treated and refractory to one IMiD (lenalidomide), one PI (bortezomib), and the most recent regimen defined by domestic drug accessibility. Daratumumab had not been approved in China when MARCH started but was conditionally approved for marketing in China during the course of MARCH. Therefore, a number of patients who failed daratumumab were subsequently enrolled in the study. In addition, eligible RRMM patients had measurable disease based on International Myeloma Working Group (IMWG) guidelines [13], had an Eastern Cooperative Oncology Group (ECOG) [14] performance score of 0–2, and had adequate organ system function including renal, hepatic, and hematopoietic functions. All patients provided written informed consent prior to enrollment. The study was conducted in accordance with the Declaration of Helsinki and Good Clinical Practice guidelines, following approval by independent ethics committees or institutional review boards at each study site. This trial is registered at ClinicalTrials.gov, NCT03944057.

### Treatment

Patients received selinexor 80 mg combined with dexamethasone 20 mg orally on day 1 and day 3 of each week, in 4-week cycles until progressive disease (PD), death, unmanageable toxicity, or withdrawal of consent, whichever occurred first.

### Endpoints and assessment

The primary endpoint for the study was the overall response rate (ORR) per IMWG as determined by an independent review committee (IRC). Secondary endpoints included overall survival (OS), progression-free survival (PFS), time to response (TTR), duration of response (DOR), safety, and PK profile.

Response assessment was performed every 28 days on day 1 of each cycle. Adverse event (AE) was assessed using the NCI Common Terminology Criteria for Adverse Events (CTCAE) version 4.03. Blood sampling (3 mL per sampling point) was collected on day 1 (single-dose) and day 15 (multiple-dose) of cycle 1 to characterize the selinexor PK profile. Plasma concentrations were determined at the following distinct time points: pre-dose, 0.5 h, 1 h, 1.5 h, 2 h, 3 h, 4 h, 5 h, 6 h, 8 h, 10 h, 24 h, and 48 h. Pharmacokinetics ethnicity sensitivity analysis was performed to evaluate the potential impact of ethnic factors on the acceptability and difference in the data of Chinese and western patients [15–19].

### Statistical method

Patients who received at least 1 dose of study treatment (modified intent-to-treat [mITT]/safety population) were used for efficacy and safety analysis. Demographics, baseline disease characteristics, and prior anti-MM therapies were summarized for the mITT populations. For the primary analysis of ORR, the primary endpoint, the sample size of 82 patients would provide 80% power to detect a statistical significance with the target ORR of 28% against the threshold ORR (i.e., null hypothesis) of 15% (one-sided *α*=0.025); statistical significance would be declared if the lower bound of the 95% confidence interval (CI) would be greater than 15%. Time to event endpoints including PFS, OS, and DOR were analyzed using the Kaplan-Meier method.

Efficacy was also evaluated in the specified subgroups, including triple-class refractory and triple-class exposed, as well as baseline age, creatinine clearance, MM subtype, revised-International Staging System (R-ISS) stage, high-risk cytogenetic abnormality by FISH, prior therapy, and the number of regimens.

Plasma sample analysis was performed using validated high-performance liquid chromatography tandem mass spectrometry method to determine the blood concentration of selinexor. PK parameters were obtained by analyzing adequate plasma drug concentration data and calculated using the NCA model with Phoenix WinNonlin v8.3.1. The descriptive statistics of PK parameters were summarized using SAS 9.4.

## Results

### Patients

**Fig. 1:**
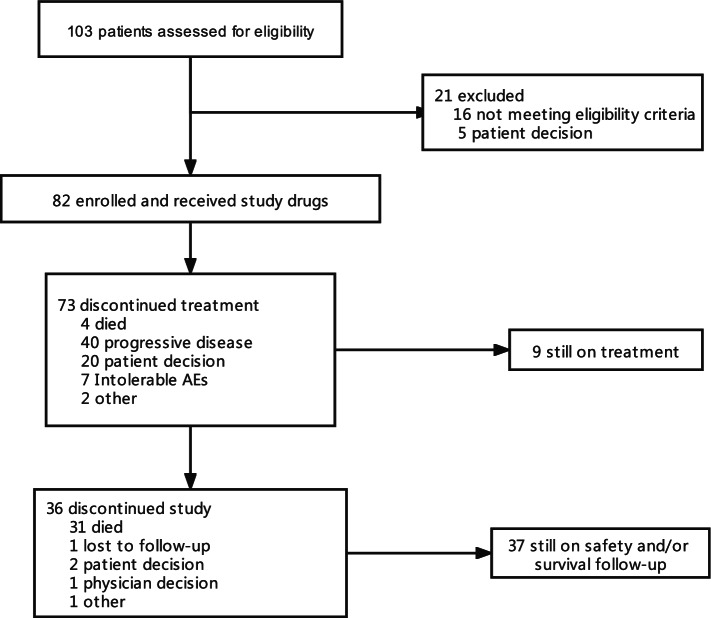
Patient disposition

Between August 2019 and February 2021, 103 patients were assessed for eligibility for the study. A total of 82 patients were enrolled and received study treatment. As of the cut-off date of May 06, 2021, 9 patients were still receiving study treatment, and 37 patients were still on safety or survival follow-up (Fig. 1). Seventy-three patients (89.0%) discontinued study treatment. The main reasons for that included disease progression (48.8%), patients’ decision (24.4%), and adverse events (8.5%). All 82 patients were included in the mITT or safety population for efficacy and safety analyses.

Baseline demographics and disease characteristics are summarized in Table 1. Patients ranged from 42 to 82 years in age, with a median of 60.0 years. The median duration from initial diagnosis was 3.2 years (range 0.2–13.4). Most patients (72%) had baseline R-ISS stage II/III. A total of 55 patients (67.1%) had high-risk cytogenetic abnormalities with 22.0% del (17p13), 25.6% t(4;14), 6.1% t(14;16), and 64.6% 1q amplification. Eighteen patients (22.0%) had baseline plasmacytoma and 18 patients (22.0%) had creatinine clearance < 60 mL/min.

**Table 1:** Baseline demographics and disease characteristics

Baseline characteristics	mITT population (***N*** = 82)
Median age, years (range)	60.0 (42, 82)
Patient age, *n* (%)	
≥65	32 (39.0)
Gender, *n* (%)	
Male	43 (52.4)
Female	39 (47.6)
Baseline ECOG performance status, *n* (%)	
0	26 (31.7)
1	54 (65.9)
2	2 (2.4)
Tumor burden increase (screening to pre-dose), *n* (%)	60 (73.2)
Median, %	23.1
Range, %	2.1–317.9
MM subtype at baseline, *n* (%)	
IgG	40 (48.8)
IgA	17 (20.7)
IgD	7 (8.5)
Others	19 (23.2)
R-ISS stage at baseline, *n* (%)	
I	23 (28.0)
II	50 (61.0)
III	9 (11.0)
High-risk cytogenetic abnormalities	55 (67.1)
del 17p13	18 (22.0)
t(4;14)	21 (25.6)
t(14;16)	5 (6.1)
1q amplification	53 (64.6)
Creatinine clearance, mL/min, *n* (%)	
<30	3 (3.7)
30–<60	15 (18.3)
≥60	64 (78.0)
Median time since initial MM diagnosis, years (range)	3.2 (0.2–13.4)
Respond to last regimen (≥PR), *n* (%)	24 (29.3)
Median prior regimens (range)	5 (1–16)
Prior therapy, *n* (%)	
Lenalidomide	
Exposed vs refractory	82 (100.0) vs 82 (100.0)
Bortezomib	
Exposed vs refractory	82 (100.0) vs 82 (100.0)
Daratumumab	
Exposed vs refractory	23 (28.0) vs 20 (24.4)
Ixazomib	
Exposed vs refractory	28 (34.1) vs 26 (31.7)
Carfilzomib	
Exposed vs refractory	6 (7.3) vs 5 (6.1)
Pomalidomide	
Exposed vs refractory	10 (12.2) vs 9 (11.0)
Prior ASCT	18 (22.0)
Prior CAR-T	10 (12.2)

*ASCT* Autologous stem cell transplantation, *CAR* Chimeric antigen receptor, *ECOG* Eastern Cooperative Oncology Group, *mITT* Modified intent-to-treat, *MM* Multiple myeloma, *R-ISS* Revised-International Staging System

Patients enrolled had highly refractory disease and were heavily pretreated. The median number of prior regimens was 5 (range 1–16). Among 82 patients, 18 patients (22.0%) received autologous stem cell transplantation (ASCT) and 10 patients (12.2%) had prior CAR-T therapy. All 82 patients (100%) were documented refractory to lenalidomide and bortezomib, of whom 20 patients (24.4%) were also refractory to daratumumab. These 20 patients constituted the triple-class refractory population, with the median prior regimen number of 6 (range 1–14).

### Efficacy

**Fig. 2:**
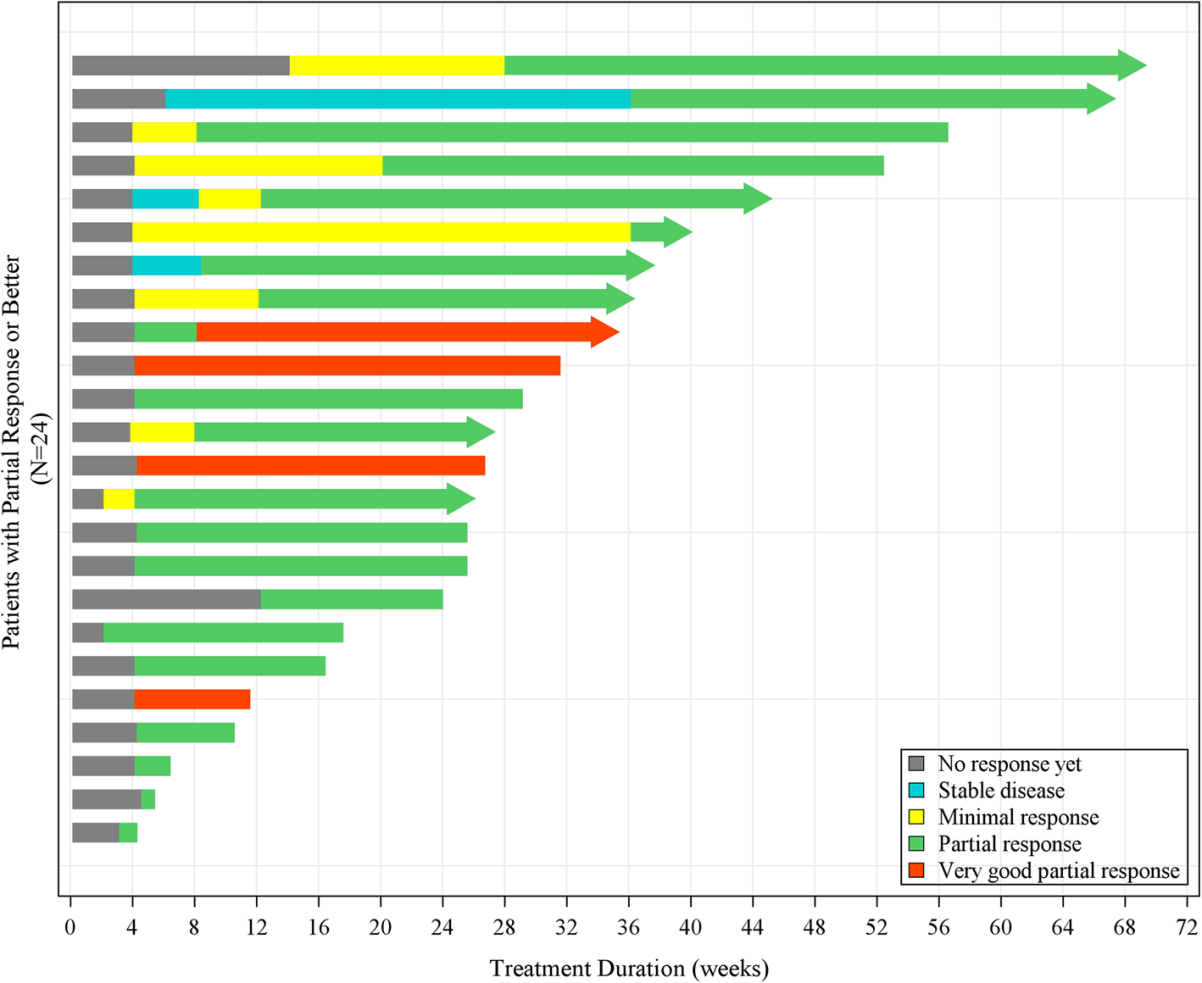
Duration of response/treatment by individual responders. An arrow at the end indicates the treatment was ongoing as of data cut-off

The primary efficacy endpoint for the study was ORR per IMWG by an IRC. The ORR in the mITT population was 29.3% (95% *CI* 19.7, 40.4), which included 4 (4.9%) patients with VGPR and 20 (24.4%) patients with PR. The lower limit of the 95% CI for ORR (19.7%) was greater than the pre-defined threshold of 15%, confirming that the primary endpoint had been reached and that the ORR of 29.3% could be considered to have demonstrated statistically significant efficacy of the Sd regimen (*p* = .0001). The median time to PR or better was 1 month (range 0.5–8.4), and the median duration of response (DOR) was 4.7 months (95% CI 2.02, not estimable [NE]) (Table 2). Figure 2 illustrates the duration of response for all responders. It was observed that a deeper response could be obtained with a longer treatment duration.

**Table 2:** Summary of best overall response

	mITT population (***N*** = 82)
ORR, *n* (%)	24 (29.3)
95% *CI*	19.7, 40.4
VGPR, *n* (%)	4 (4.9)
PR, *n* (%)	20 (24.4)
MR, *n* (%)	8 (9.8)
SD, *n* (%)	37 (45.1)
PD, *n* (%)	7 (8.5)
Not estimable, *n* (%)	6 (7.3)

*ORR*, the percentage of patients achieving confirmed best response of sCR, CR, VGPR, or PR

*CI* Confidence interval, *mITT* Modified intent-to-treat, *MR* Minimal response, *ORR* Overall response rate, *PD* Progressive disease, *PR* Partial response, *SD* Stable disease, *VGPR* Very good partial response

**Fig. 3:**
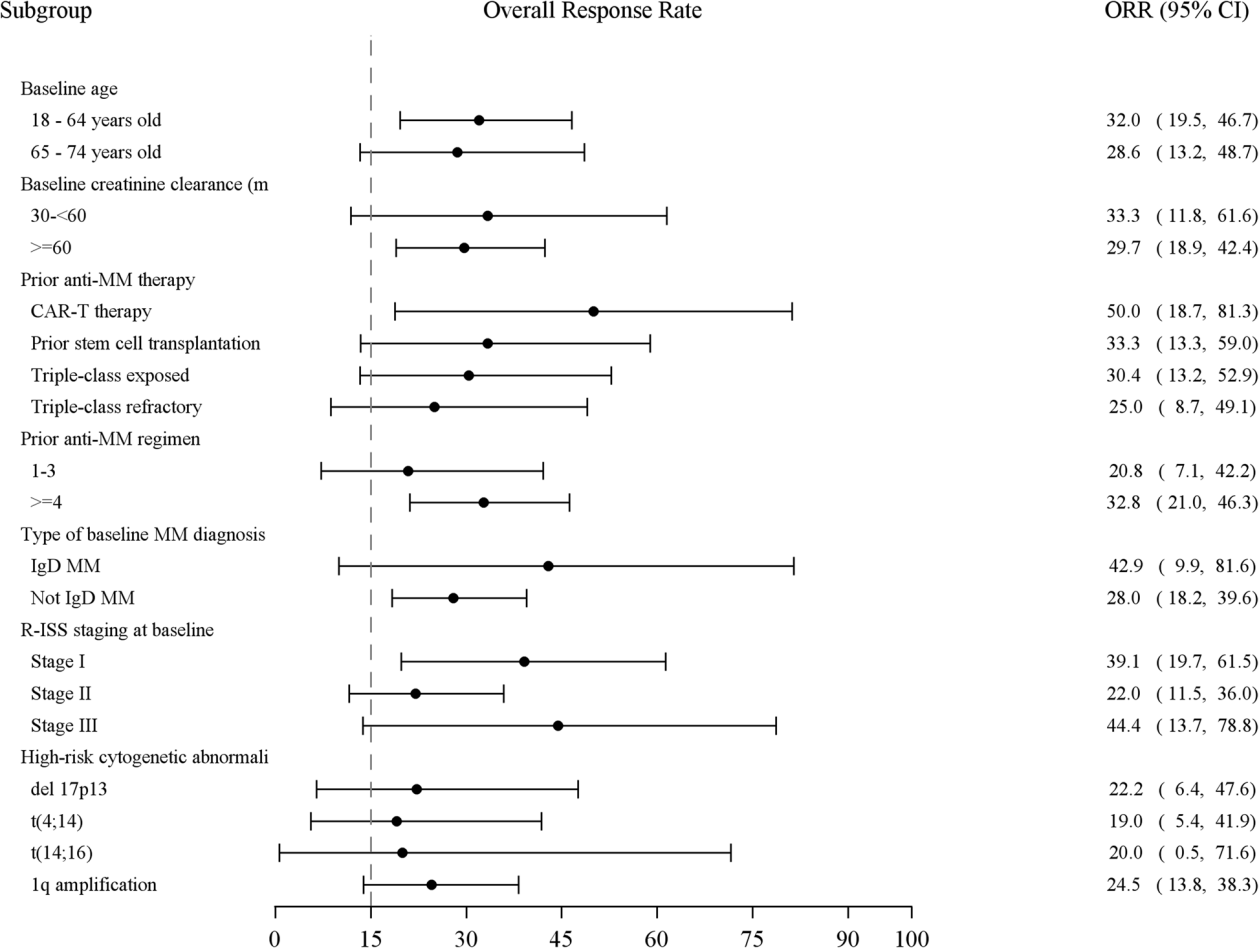
Subgroup analysis of ORR. The dotted line indicates the null hypothesis of ≤15% for the ORR reported in this study

A subgroup analysis for ORR was also performed (Fig. 3). It is noted that both triple-class refractory and triple-class exposed patients could benefit from the Sd regimen, with ORR of 25.0% and 30.4%, respectively. ORR was 25.5% in patients with high-risk cytogenetic abnormalities, including 22.2% in those with del 17p13. In addition, ORR was 28.6% in elderly patients (65–74 years), 44.4% in those with baseline R-ISS III, 42.9% in IgD MM, 33.3% in those with baseline creatinine clearance 30–<60 mL/min, 32.8% in those with prior regimen number ≥ 4, and 33.3% in those with prior stem cell transplant. In summary, the response rate was generally consistent across prespecified subgroups.

Of note, ORR was 50.0% in the 10 patients who had prior CAR-T therapy, with the median duration of response of 1.4 months. As expected, these patients had a longer disease history and received more prior regimens than the total population. The median duration from MM’s initial diagnosis was 5.2 years, and the median number of prior regimens was 9.5. Five patients experienced very rapid disease progression as indicated by a median of 46.2% increase of tumor burden from screening to C1D1. The median PFS was 1.9 months and median OS was not reached with the estimated 12-month OS rate of 68.6%. AEs reported in this subgroup were consistent with those reported in the overall population.

**Fig. 4:**
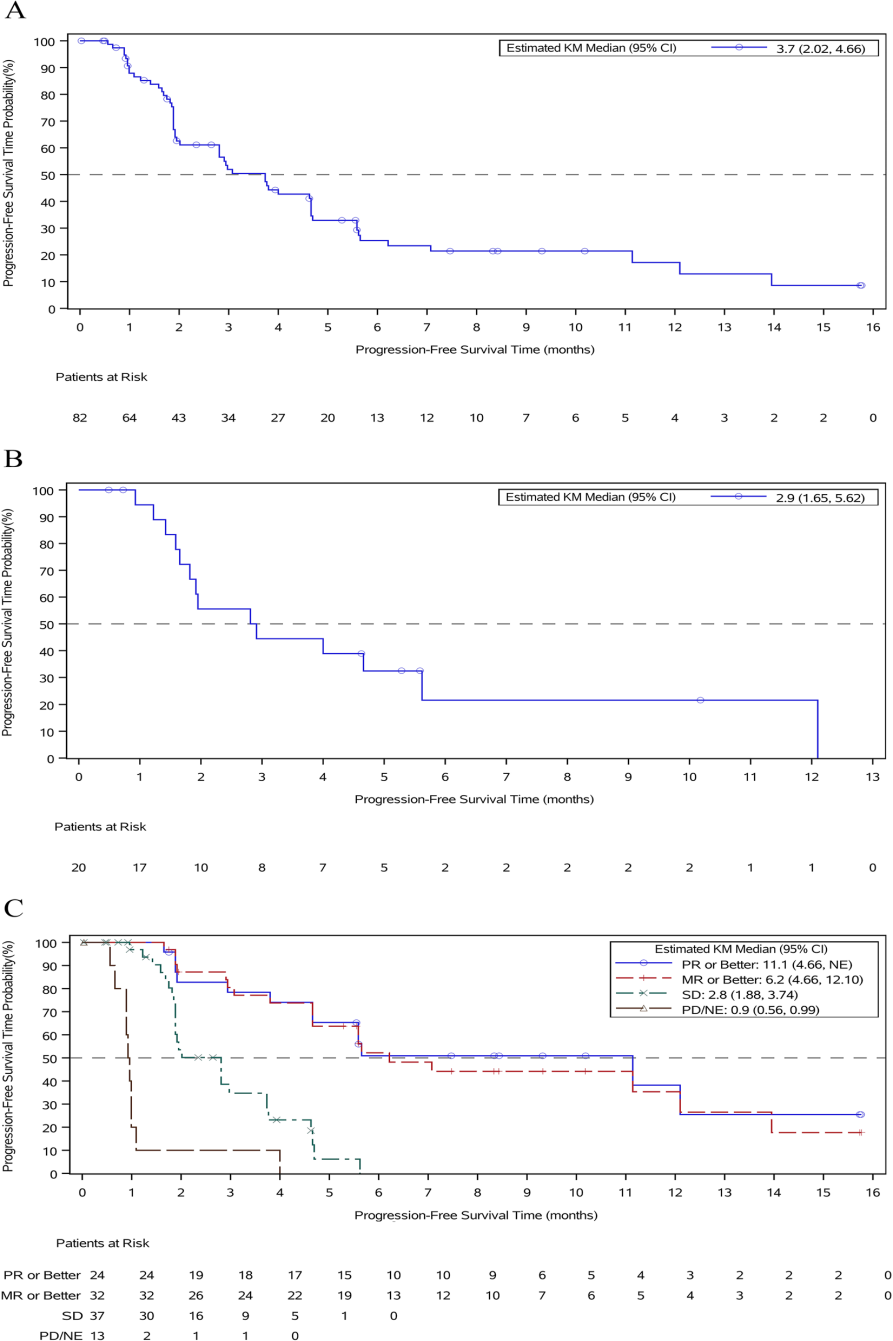
Progression-free survival (PFS) A Kaplan-Meier curve of PFS in the modified intent-to-treat population. B Kaplan-Meier curve of PFS in the triple-class refractory population. C Kaplan-Meier curve of PFS by best response

As of the cut-off date, with a median follow-up of 10.6 months, the median PFS in the mITT population was 3.7 months (95% *CI* 2.02, 4.66). The median PFS in the triple-class refractory population was 2.9 months (95% *CI* 1.65, 5.62). For patients who had a PR or better, the median PFS was 11.1 months (95% *CI* 4.66, NE) (Fig. 4).

**Fig. 5:**
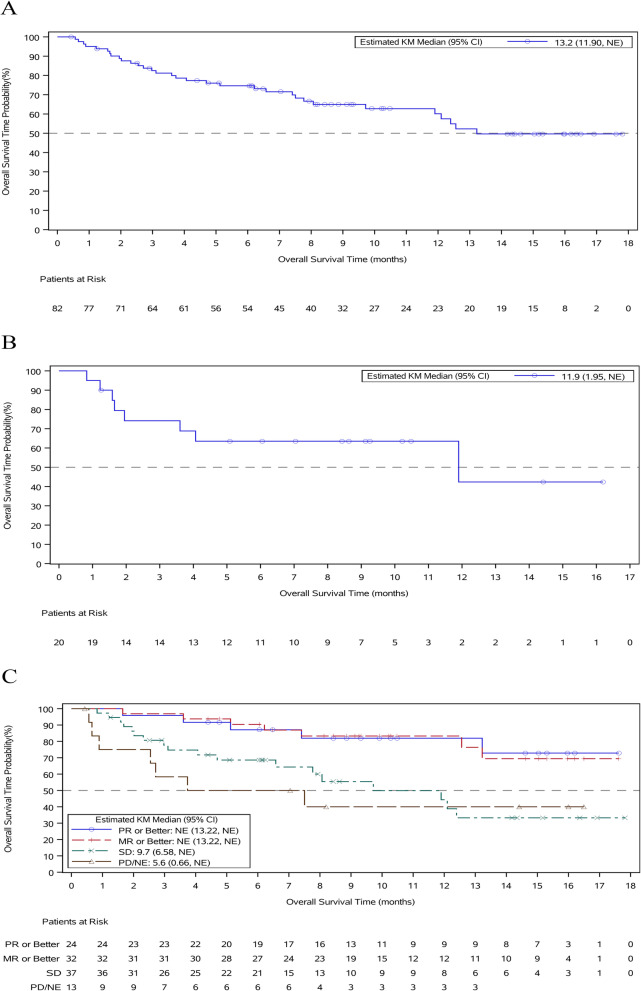
Overall survival (OS) A Kaplan-Meier curve of OS in the modified intent-to-treat population. B Kaplan-Meier curve of OS in the triple-class refractory population. C Kaplan-Meier curve of OS by best response.

The median OS in the mITT population was 13.2 months (95% *CI* 11.90, NE). The median OS in the triple-class refractory population was 11.9 months (95% *CI* 1.95, NE). For patients who had an MR or better, the median OS was not reached (95% *CI* 13.22, NE); the estimated 1-year survival rate was 60.1% (95% *CI* 46.9, 71.0) (Fig. 5).

### Exposure and safety

The median treatment duration of Sd was 13.7 weeks (range 1.1–70.0). 54.9% of patients received treatment ≥12 weeks and 28% of patients ≥24 weeks. The median weekly dose received was 113.7 mg (range 55.4–160.0). The majority of patients had dose modifications of selinexor, including dose interruptions in 75.6% of patients and dose reductions in 84.1% of patients. Among the patients with dose reductions, the median number of dose reductions was 2; 33 patients (40.2%) had a reduction to 100–120 mg weekly and 17 patients (20.7%) had a reduction to 80 mg weekly. The median time to the first dose reduction was 22.0 days (range 4–120). The median treatment duration was 16.6 weeks (range 2–70) for the patients who had dose reduction vs 4.3 weeks (range 1–27) for those who did not have. Eight patients re-escalated the dose as prespecified after AE was stabilized for more than 4 weeks.

In terms of treatment-emergent adverse event (TEAE), overall, all 82 patients had at least one TEAE. The most common (≥50%) TEAEs included thrombocytopenia (87.8%), anemia (86.6%), leukopenia (82.9%), nausea (78.0%), lymphopenia (76.8%), neutropenia (72.0%), hyponatremia (67.1%), weight decreased (65.9%), decreased appetite (62.2%), asthenia (59.8%), hyperglycemia (56.1%), and vomiting (50.0%). The most frequent grade 3/4 TEAE (>10%) were anemia (57.3%), thrombocytopenia (51.2%), lymphopenia (42.7%), neutropenia (40.2%), hyponatremia (29.3%), lung infection (26.8%), hypokalemia (12.2%), and asthenia (9.8%)/fatigue (2.4%) (Table 3). The most common adverse events leading to dose interruption or reduction were thrombocytopenia, neutropenia, and lung infection.

**Table 3:** Most common adverse events by CTCAE grade (≥20%)

Preferred term	mITT population (***N*** =82)				
	Grade 1/2	Grade 3	Grade 4	Grade 5	All grades
**Hematologic TEAEs,** ***n*** **(%)**					
Thrombocytopenia	30 (36.6)	23 (28.0)	19 (23.2)	0	72 (87.8)
Anemia	24 (29.3)	43 (52.4)	4 (4.9)	0	71 (86.6)
Leukopenia	36 (43.9)	27 (32.9)	5 (6.1)	0	68 (82.9)
Lymphopenia	28 (34.1)	24 (29.3)	11 (13.4)	0	63 (76.8)
Neutropenia	26 (31.7)	20 (24.4)	13 (15.9)	0	59 (72.0)
**Non-hematologic TEAEs,** ***n*** **(%)**					
Nausea	58 (70.7)	6 (7.3)	0	0	64 (78.0)
Hyponatremia	31 (37.8)	24 (29.3)	0	0	55 (67.1)
Weight decreased	54 (65.9)	0	0	0	54 (65.9)
Decreased appetite	48 (58.5)	3 (3.7)	0	0	51 (62.2)
Asthenia	41 (50.0)	8 (9.8)	0	0	49 (59.8)
Hyperglycemia	38 (46.3)	7 (8.5)	1 (1.2)	0	46 (56.1)
Vomiting	35 (42.7)	6 (7.3)	0	0	41 (50.0)
Hypocalcemia	30 (36.6)	5 (6.1)	1 (1.2)	0	36 (43.9)
Hypokalemia	21 (25.6)	9 (11.0)	1 (1.2)	0	31 (37.8)
Hypoalbuminemia	25 (30.5)	1 (1.2)	0	0	26 (31.7)
Diarrhea	22 (26.8)	3 (3.7)	0	0	25 (30.5)
Lung infection	3 (3.7)	20 (24.4)	1 (1.2)	1 (1.2)	25 (30.5)
Hypophosphatemia	14 (17.1)	5 (6.1)	0	0	19 (23.2)
Aspartate aminotransferase increased	17 (20.7)	0	2 (2.4)	0	19 (23.2)
Alanine aminotransferase increased	15 (18.3)	2 (2.4)	1 (1.2)	0	18 (22.0)
Insomnia	18 (22.0)	0	0	0	18 (22.0)

When multiple adverse events occurred in the same patient, the highest CTCAE grade was used for statistics; when the same patient had multiple adverse events under the same SOC or PT, the highest CTCAE grade was used for statistics

Most grade 3/4 TEAEs were hematologic events, which could be closely monitored and well managed through routine blood tests, dose modification, and supportive treatment, such as blood transfusion, growth factor, or platelet stimulating factor. For example, 72.2% of patients with thrombocytopenia and 57.6% of patients with neutropenia/febrile neutropenia received supportive treatment. The incidence of grade 3/4 thrombocytopenia was comparable with that of other selinexor studies and not associated with severe hemorrhagic events. Less than 10% of patients experienced hemorrhagic events within 5 days of thrombocytopenia, with the majority (>70%) being grade 1/2. Only 3 patients discontinued treatment due to the events. Most non-hematologic TEAEs were gastrointestinal or constitutional events, predominantly limited to grade 1/2 severity and reversible through dose modification and supportive treatment. Multiple non-hematologic TEAEs could occur at the same time and correlated with each other. Treatment for nausea and vomiting was critical for successful safety management, which simultaneously improved decreased appetite, weight decrease, and asthenia/fatigue. Thus, patients were requested to receive prophylactic antiemetic and appetite enhancing therapy, including 5-HT3 receptor antagonist, NK1 receptor antagonist, appetite stimulant, and/or olanzapine. The mechanism of hyponatremia remains unknown, although it was often seen in association with loss of body fluid and/or imbalance of electrolytes. The incidence of grade 3 hyponatremia was 29.3%, but the majority of the patients had serum sodium levels between 125 and 130 mmol/L with no obvious symptoms. Laboratory monitoring is necessary, and the events were rapidly controlled with sodium supplementation. The incidence of lung infection in the study was expected. Lung infection is a common complication in patients with hematological tumors, especially in elderly patients with myeloma who have received multiple lines of therapy. At baseline, about 50% of the patients complicated with underlying respiratory diseases (including chronic bronchitis and chronic obstructive pulmonary disease). Most of the events improved with anti-infection treatment. The incidence of the events leading to treatment discontinuation was only 2.4%.

Serious adverse events (SAEs) were reported in 45 (54.9%) patients, with lung infection and thrombocytopenia (14.6% each) as the most common SAE. Grade 5 AEs occurred in 7 patients (8.5%), including infective exacerbation of bronchiectasis, sudden death, pulmonary embolism, lung infection, sepsis, and intracranial hemorrhage.

Most of TEAEs occurred in the first 2 cycles, and the incidence would decrease afterwards. A summary of incidence by study treatment period for the TEAEs of special interest is also provided in Table 4.

**Table 4:** Summary of TEAEs of special interest—incidence by time periods

Preferred term	Study treatment period		
	1–8 weeks	9–24 weeks	>24 weeks
# of subjects continuing treatment, *n* (%)	82 (100)	58 (100)	23 (100)
Thrombocytopenia	70 (85.4)	42 (72.4)	18 (78.3)
Nausea/vomiting	68 (82.9)	21 (36.2)	5 (21.7)
Fatigue/asthenia	58 (70.7)	15 (25.9)	5 (21.7)
Neutropenia/febrile neutropenia	54 (65.9)	34 (58.6)	10 (43.5)
Hyponatremia	52 (63.4)	18 (31.0)	6 (26.1)
Decreased appetite	46 (56.1)	16 (27.6)	8 (34.8)
Weight decreased	44 (53.7)	27 (46.6)	7 (30.4)
Neurotoxicity	24 (29.3)	7 (12.1)	1 (4.3)
Diarrhea	22 (26.8)	7 (12.1)	1 (4.3)
Pneumonia	20 (24.4)	7 (12.1)	3 (13.0)
Vision blurred	5 (6.1)	2 (3.4)	2 (8.7)
Cataract	1 (1.2)	1 (1.7)	1 (4.3)

### Pharmacokinetics

**Fig. 6:**
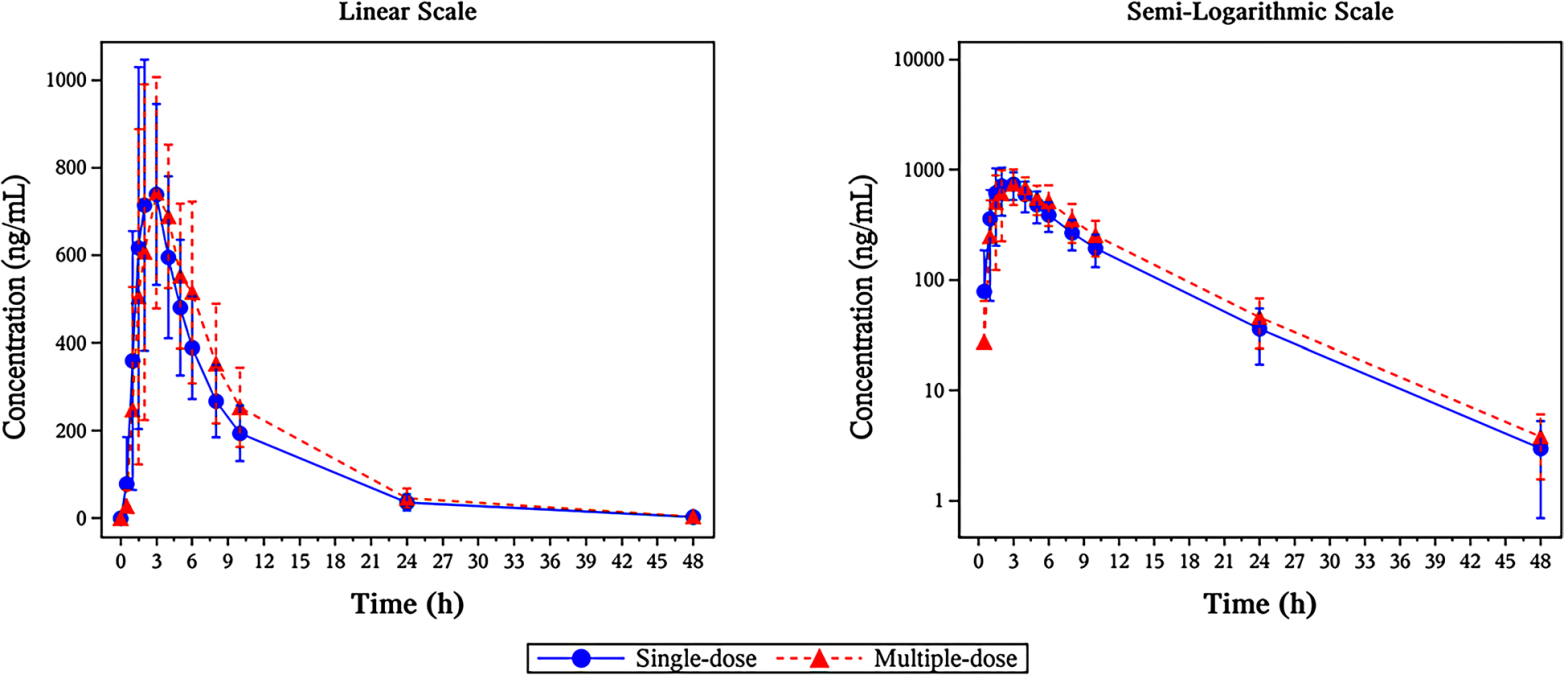
Average plasma concentration-time curve comparison after single and multiple doses

PK sample collection was performed in 18 patients, of whom 12 received multiple-dose PK sampling. Following a single dose of selinexor 80 mg on C1D1, selinexor PK profile (Fig. 6) was characterized by rapid absorption with median *T*_max_ of 2.88 h, mean *t*_1/2_ of 6 h, mean *C*_max_ of 862 ng/mL, and mean AUC_0–48h_ of 5804 h ng/mL. PK results on C1D15 were similar to C1D1, and *R*_ac(Cmax)_ and *R*_ac(AUC)_ were 1.04 and 1.10, respectively, indicating no significant accumulation following twice-weekly administration. No significant differences were found in *t*_1/2_, AUC_0–*t*_, *T*_max_, CL/F, and V_d_/F between single dose and multiple dose. No significant inter-individual variability was seen as evidenced by the geometric mean percent coefficient of variation range for AUC_0–∞_ (30.7–32.6%) and *C*_max_ (22.0–35.3%).

Comparing PK parameters obtained from MARCH and five global selinexor studies [15–19], *T*_max_ and *t*_1/2_ were similar, with median *T*_max_ of 2 to 2.95 h and mean *t*_1/2_ of 5.84 to 6.24 h; *C*_max_, AUC_0–t_, and AUC_0–inf_ were slightly higher in MARCH, but considering inter-individual variation, it generally showed an overlapping distribution within these studies (Supplementary 1). There was no significant ethnic difference observed between Chinese and western populations.

## Discussion

The Sd regimen induced clinically meaningful responses in MARCH with ORR of 29.3% in RRMM patients. The median PFS and OS were 3.7 and 13.2 months, respectively, suggesting a potentially prolonged clinical benefit. The efficacy was consistent with that seen in STORM (ORR 26.2%, median PFS 3.7 months, median OS 8.4 months). As the China regulatory bridging study for STORM, MARCH further verified the efficacy and safety of Sd in Chinese RRMM patients. Although inclusion criteria were not exactly the same between MARCH and STORM due to differences in domestic drug availability, patients enrolled in MARCH remained a group with multiple poor prognostic factors. During the screening period, 73.2% of patients had a rapid increase in tumor burden with a median increase of 23.1%. The overall survival of such individuals is often dismal. With the results of MARCH, the Sd regimen offers a valuable treatment option for these patients.

Subgroup analysis demonstrated that the efficacy of selinexor plus dexamethasone was consistent across different subgroups, including triple-class refractory RRMM patients who obtained an ORR of 25.0% and median DOR of 10.2 months. Based on the MARCH and the STORM data, Sd has been granted conditional approval by the Chinese National Medical Products Administration (NMPA) for the treatment of RRMM patients who have received at least one PI, one IMiD, and an anti-CD38 monoclonal antibody on 14 December 2021.

There were more patients with prior CAR-T therapy in MARCH than in STORM (10 vs 2 patients), reflecting the current clinical practice in recent years. The 10 patients in MARCH exhibited a promising signal with an ORR of 50% and a manageable safety profile. It showed that patients refractory to available MM therapies could respond to a drug with a new mechanism of action. However, caution should be exercised when interpreting the data considering the small sample size and relatively short duration of response. A more intensive selinexor-based regimen may be worth further exploration.

Renal function abnormality is one of the most common MM complications. Previous population PK analysis indicated that mild, moderate, or severe renal impairment was not expected to change the PK profile of selinexor. Therefore, patients with creatinine clearance ≥ 20 mL/min were allowed to be enrolled, and response outcomes confirmed that drug efficacy was not affected by baseline renal function. Considering a higher proportion of light chain MM and IgD MM, and a generally later diagnosis in this geography, selinexor with dexamethasone emerges as an appealing treatment option for MM patients with moderate to severe renal dysfunction.

In addition, IgD MM is a very rare subtype of MM, accounting for only 1–2% of MM according to global studies. However, it accounts for about 6–8% of Chinese MM based on some retrospective studies [20, 21]. IgD MM has unique disease characteristics, with a higher incidence of anemia, hypercalcemia, renal impairment, elevated lactate dehydrogenase, extramedullary plasmacytoma, and bone lesions. It has been reported that the median survival time of IgD MM was lower than patients with other MM diagnosis types [22]. IgD MM in the MARCH study accounted for 8.5% of the patient population, which was consistent with the relatively higher incidence in China. An ORR of 42.9% was obtained in IgD MM patients in this study, indicating the potential advantage of Sd in a Chinese MM population in whom the incidence of IgD MM is relatively high.

Elderly patients (≥65 years) could derive similar benefit from the study treatment to the general study population, with an ORR of 25.0%. Considering MM is a disease predominantly in the elderly, the oral treatment regimen is helpful to improve treatment compliance for elderly patients. Better supportive care might be needed to prolong the treatment duration for clinical benefit.

The safety results of the MARCH study were consistent with those observed in other selinexor studies, including the STORM study. The most common events were thrombocytopenia and gastrointestinal and constitutional events. These events were reversible and could be well managed by dose modification and prophylactic/supportive treatment. No new safety signals were identified. Although the majority of patients (84.1%) underwent dose reduction, the higher starting dose seemed necessary to sufficiently control the disease in this heavily pretreated MM population with a significant disease burden. These patients had a narrow window of time to achieve disease control in order to minimize and prevent end-organ damage. It was also observed that patients who had selinexor reduction had a considerably longer treatment duration compared with those without selinexor reduction (16.6 vs 4.3 weeks). These observations suggest that active dose modification may improve the tolerability of selinexor, thereby prolonging treatment duration and extending the clinical benefit. The trend of AEs by time period also revealed that the incidence rate peaked in the first 2 months then decreased significantly. Close monitoring and management of AEs is particularly recommended during the first 2 months of treatment.

It was observed that the incidence and severity of adverse events were generally consistent between MARCH and STORM. The incidence of all-grade hematological AEs was higher in MARCH than STORM, although ≥ grade 3 hematological AE rates were very similar; Similarly, the incidence of all-grade metabolic-relevant AEs was also higher in MARCH than STORM, including hyponatremia, hyperglycemia, hypocalcemia, hypokalemia, and hypoalbuminemia. All these AEs were reported as a consequence of abnormal laboratory results. To investigate the difference, we further compared laboratory results between the two studies. There was no significant difference in laboratory abnormalities, including complete blood counts, electrolytes, and blood glucose levels. However, the percentage of laboratory abnormality reported as TEAE by investigators in the MARCH study was higher than the STORM study. This appears to be a more conservative assessment of such events by Chinese investigators in this single-arm study.

Finally, it is also worth noting the high incidence rate of lung infection in MARCH. It has been reported that the risk of lung infection in MM patients is 7.7 times higher than in healthy populations, and infection is one of the main reasons for death in MM patients [23]. Multiple factors may predict the occurrence and prognosis of lung infection, including the low level of immunoglobulin, age, immune function, baseline lung function, and the long-term use of cytotoxic drugs and glucocorticoids. Considering the baseline respiratory disease prior to study treatment and without concurrent neutropenia, a substantial part of lung infection events could be reasonably considered to be more associated with patients’ underlying diseases.

Pharmacokinetic analysis results showed that selinexor was absorbed quickly after administration with a median *T*_max_ of 3 h and a mean *t*_1/2_ of 6 h. No significant accumulation was seen with twice-weekly administration, and no significant inter-individual variability was observed. There was also no significant ethnic difference between Chinese and western populations. These data are consistent with the belief that the dose regimen of selinexor at 80 mg twice weekly plus low-dose dexamethasone is an appropriate selection for Chinese patients with MM.

The limitation of the present study is that this is a single-arm, bridging study with a relatively limited sample size, especially only a small group of triple-class-refractory patients being recruited, since daratumumab had not been available in China when MARCH initiated until the study had been carried out for nearly half year. On the other hand, it is the first reported study of an oral regimen with meaningful efficacy in treating double- or triple-refractory Chinese RRMM patients to date. Whereas another single-arm study in Chinese RRMM patients [24], of carfilzomib and dexamethasone (Kd), achieved an ORR of 35.8%, PFS of 5.6 months and OS of 16.6 months, patients in this Kd study were refractory to one PI and/or one IMiD, with 74% of patients being double-refractory, a median of 4 prior regimens, and baseline characteristics more favorable than the patient population in MARCH.

These data do suggest that selinexor with dexamethasone may provide a platform for the addition of other active agents, and encouraging data are starting to emerge with these combinations. Selinexor combined with bortezomib, carfilzomib, pomalidomide, lenalidomide, or anti-CD38 antibody are being explored in STOMP and BOSTON studies [25–29]. The ORR achieved with these triplet regimens has been encouraging, with ORR of 60–80%. Furthermore, the dosage and frequency of selinexor were significantly reduced in these triplet regimens, with doses between 60 and 100mg once a week utilized. The regimen of selinexor in combination with bortezomib and dexamethasone (selinexor 100 mg once a week) has been approved by FDA for MM patients who have received at least one prior therapy based on the BOSTON study. The registrational phase 3 randomized BENCH study (NCT04939142), selinexor in combination with bortezomib and dexamethasone versus bortezomib and dexamethasone in RRMM patients with 1–3 prior therapies, is now ongoing in China.

## Conclusion

This phase II, single-arm study has demonstrated the efficacy of selinexor in combination with dexamethasone in heavily treated, highly refractory MM patients in China with a manageable safety profile. The favorable benefit-risk profile supports that the Sd regimen provides a novel treatment option for Chinese MM patients who have exhausted available therapies.

## Supplementary Information

**Additional file 1: Supplementary 1.**GEBoxplot of individual systemic exposure of selinexor in Chinese and western populations. *ATG-010-MM-001: NCT03944057; KCP-330-001* [15, 17, 19]*: NCT01607892; KCP-330-002* [16]*: NCT01607905; KCP-330-003* [18]*: NCT01896505*.

## Data Availability

The datasets used and/or analyzed during the current study are available from the corresponding author on reasonable request.

## Abbreviations

ASCT: Autologous stem cell transplantation
AUC: Area under the concentration-time curve
CAR-T: Chimeric antigen receptor T cells
CBR: Clinical benefit rate
CI: Confidence interval
CL/F: Systemic clearance
*C*_max_: Peak concentration
CTCAE: Common Terminology Criteria for Adverse Events
CXDX: Cycle X day X
DOR: Duration of response
ECOG: Eastern Cooperative Oncology Group
FISH: Fluorescence in situ hybridization
GCR: Glucocorticoid receptor
IMiD: Immunomodulator
IMWG: International Myeloma Working Group
IRC: Independent review committee
mITT: Modified intent-to-treat
MR: Minimal response
NE: Not estimable
ORR: Overall response rate
OS: Overall survival
PD: Progressive disease
PFS: Progression-free survival
PI: Proteasome inhibitor
PK: Pharmacokinetics
PR: Partial response
R_ac_: Accumulation ratio
RRMM: Relapsed/refractory multiple myeloma
SAE: Serious adverse event
sCR: Stringent complete response
Sd: Selinexor+dexamethasone
SD: Stable disease
SINE: Selective inhibitor of nuclear export
*t*_1/2_: Elimination half-life
TEAE: Treatment-emergent adverse event
*T*_max_: Time to reach *C*_max_
TTR: Time to response
Vd/F: Apparent volume of distribution
VGPR: Very good partial response
XPO1: Exportin 1
