# Supplementary figures and images for: Selinexor plus low-dose dexamethasone in Chinese patients with relapsed/refractory multiple myeloma previously treated with an immunomodulatory agent and a proteasome inhibitor (MARCH): a phase II, single-arm study
Source: BMC Med. 2022 Apr 5;20:108. doi: 10.1186/s12916-022-02305-4 (PMC8981703; doi:10.1186/s12916-022-02305-4)

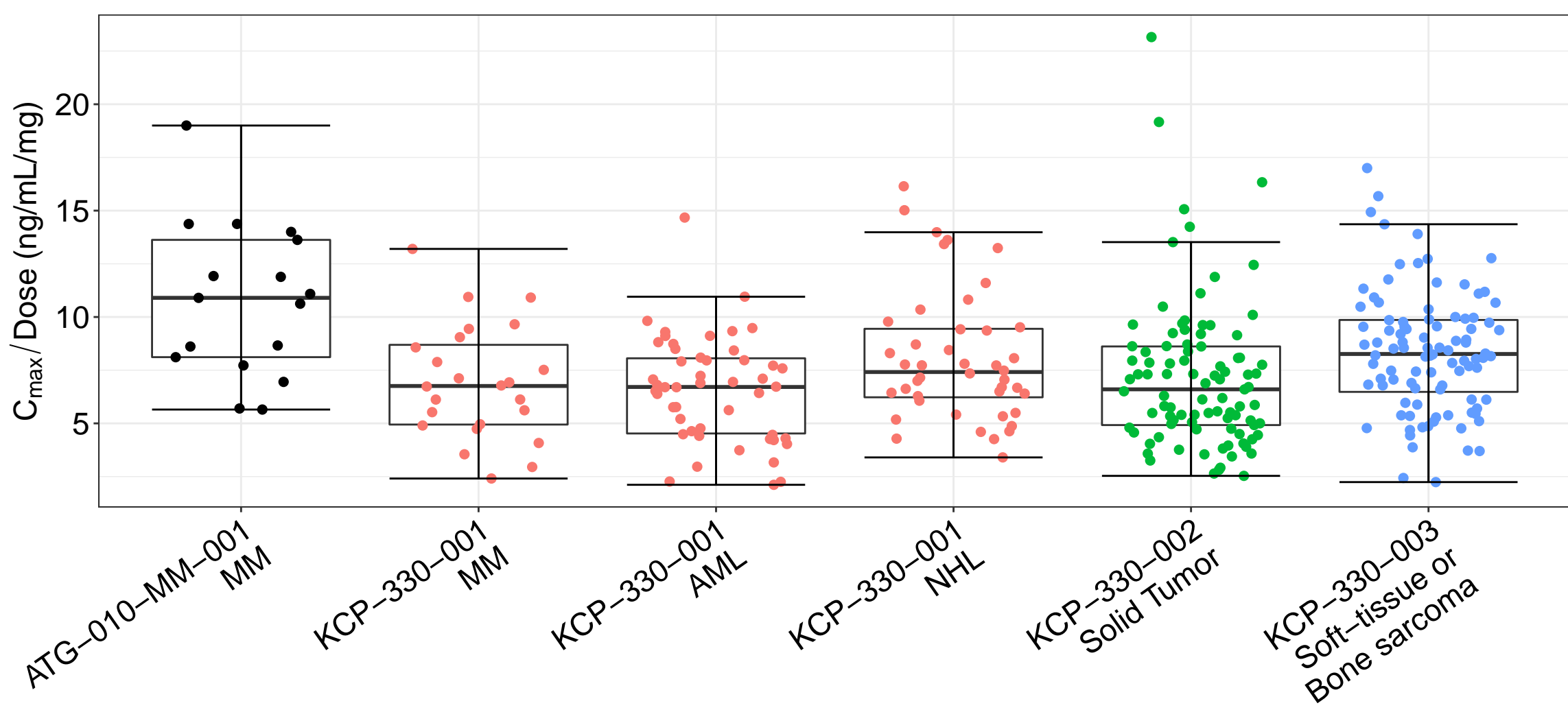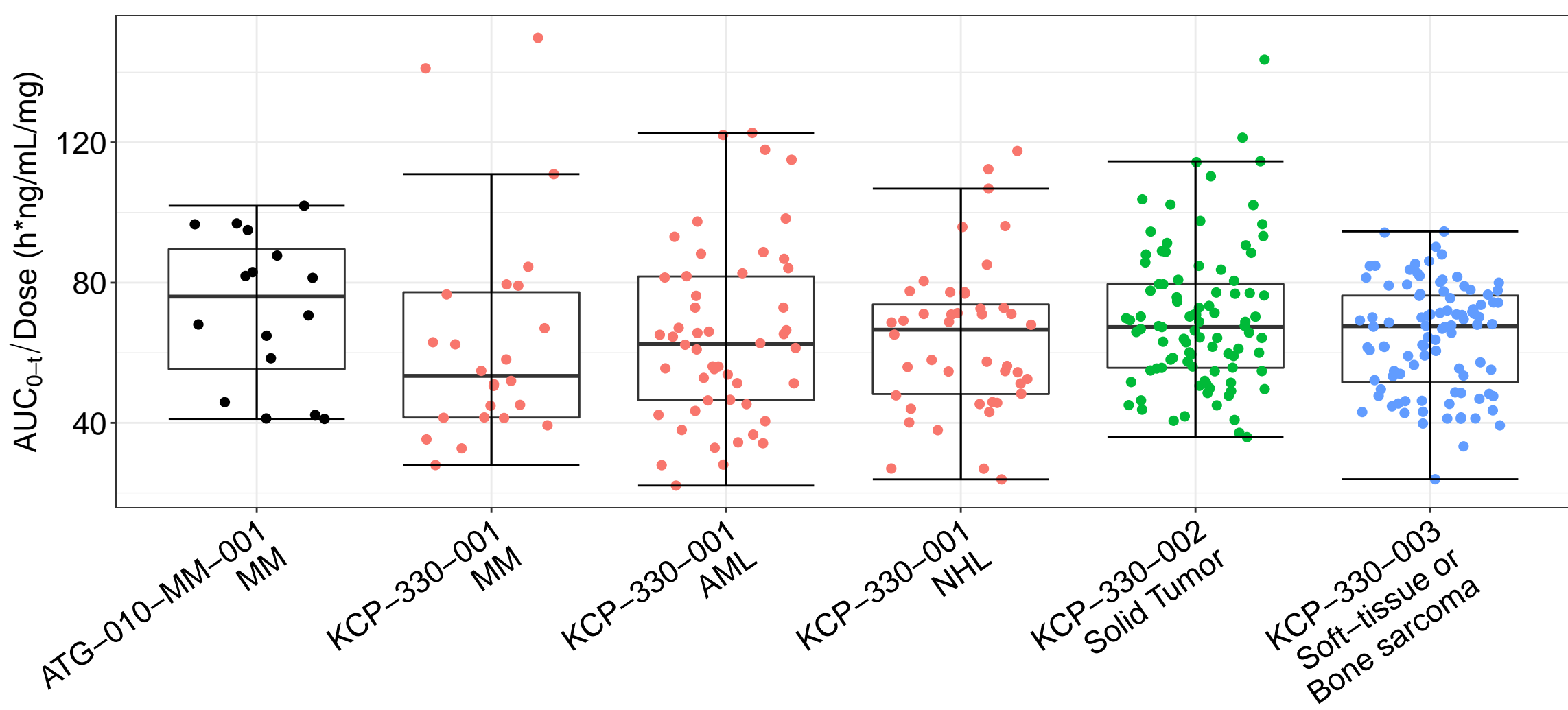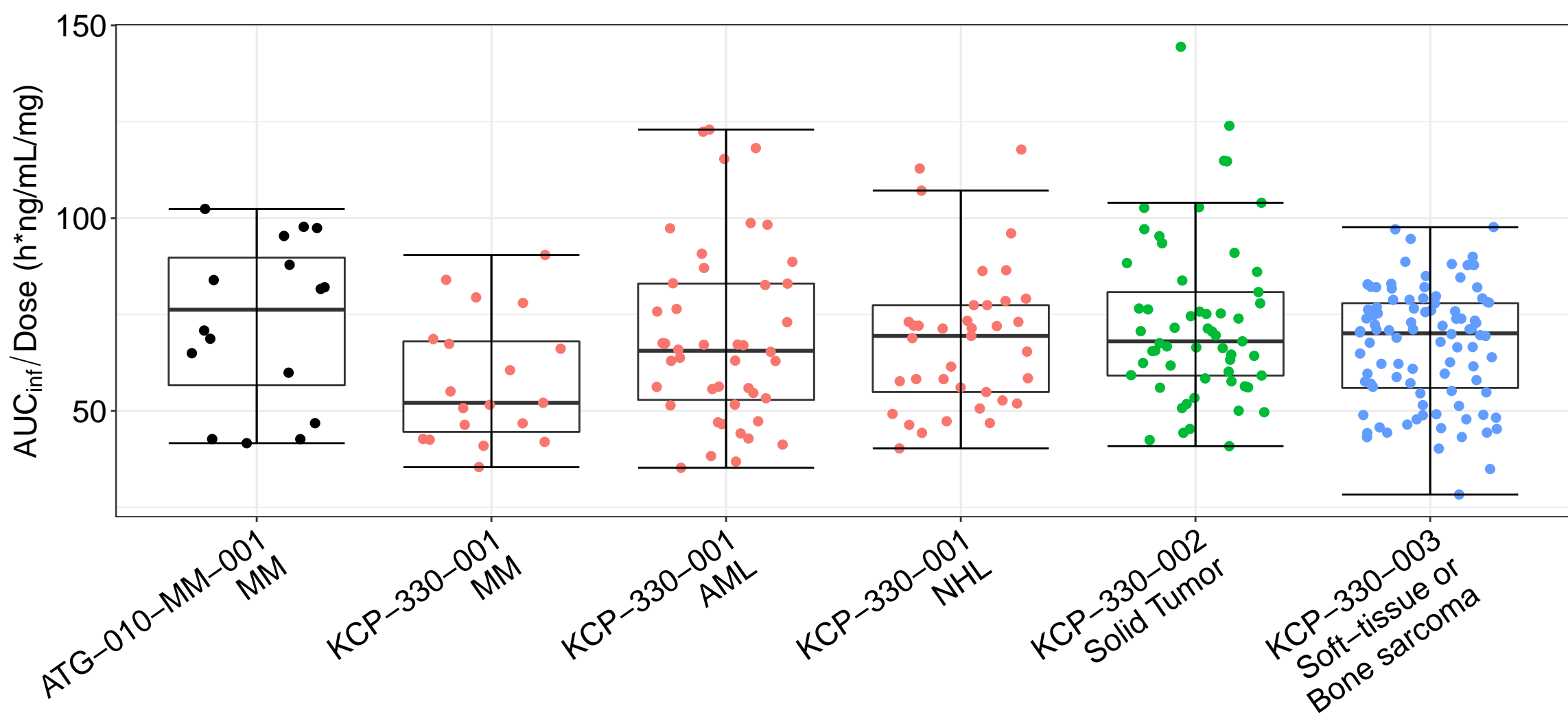

Supplement: Supplementary file 1 — Additional file 1: Supplementary 1.GEBoxplot of individual systemic exposure of selinexor in Chinese and western populations. ATG-010-MM-001: NCT03944057; KCP-330-001 [15, 17, 19]: NCT01607892; KCP-330-002 [16]: NCT01607905; KCP-330-003 [18]: NCT01896505. [file 12916_2022_2305_MOESM1_ESM.pdf]
